# Data on the activity of DNA methyltransferase in the uteri of CD-1 mice exposed to dibutyl phthalate

**DOI:** 10.1016/j.dib.2019.105061

**Published:** 2019-12-31

**Authors:** Maricarmen Colón-Díaz, Juliara Ortiz-Santana, Zelieann R. Craig

**Affiliations:** a San Juan Bautista School of Medicine, Caguas PR, USA; b School of Animal & Comparative Biomedical Sciences, University of Arizona, Tucson, AZ, USA

**Keywords:** Phthalate, Uterus, Dibutyl phthalate, Endometrium, Epigenetics, DNA methylation, DNA methyltransferase, Infertility

## Abstract

Phthalates are industrial chemicals used as plasticizers in food packaging, medical devices, and toys, as well as cosmetics used primarily by women. Epidemiological studies in women and animal studies using rodents have reported associations between phthalate exposures and adverse reproductive health outcomes. Epigenetic mechanisms are thought to be involved in the ability of environmental contaminants to influence development of disease but evidence linking exposure to phthalates and uterine DNA methyltransferase activity are lacking. This article reports the activity of DNA methyltransferase (DNMT) enzymes in uteri from CD-1 mice treated with or without dibutyl phthalate (DBP), a phthalate commonly found in the urine of women of reproductive age. CD-1 mice were orally dosed with tocopherol-stripped corn oil (vehicle) or DBP at 10 μg/kg/day, 100 μg/kg/day and 1000 mg/kg/day daily for 10, 20, and 30 days. These dosages were selected based on estimates of human intake previously reported (10 and 100 μg/kg/day) and included a high dose (1000 mg/kg/day) for comparison with classical toxicity studies. At the end of 10, 20 or 30 days of daily oral dosing, animals were euthanized within 1–2 hours after the final dose. DNMT activity was determined by subjecting uterine nuclear extracts to a commercially-available DNMT activity ELISA assay and measuring optical density with a microplate spectrophotometer at a wavelength of 450 nm. Graph Pad Prism 8 was used for data analysis to determine the activity of DNMT enzymes at different time points and doses versus vehicle. The data presented serves as a resource for researchers working in the field of toxicology because it addresses a gap in knowledge of how exposure to environmental factors such as phthalate esters could produce epigenetic alterations in the uterus, which consequently may increase the risk of developing reproductive disease.

**Table:** Specifications Table

Subject	Toxicology
Specific subject area	Epigenetics
Type of data	Tables and Graph
How data were acquired	Microplate reader (Fisher Scientific Multiscan FC)
Data format	Raw and analyzed
Parameters for data collection	On postnatal day 35, mice were pipet-fed tocopherol-stripped corn oil (vehicle control; alone or dibutyl phthalate dissolved in oil at 10 μg/kg/day, 100 μg/kg/day, and 1000 mg/kg/day. At the end of 10, 20, or 30 days of daily oral dosing, animals were sacrificed within 1–2 hours after the final dose.
Description of data collection	Nuclear extracts from mice uterus were subjected to a commercial DNMT activity ELISA assay. DNMT activity, which in this assay is proportional to the optical density measured, was detected by reading the absorbance generated by each sample using a microplate spectrophotometer at a wavelength of 450 nm.
Data source location	Caguas, Puerto Rico, USA
Data accessibility	Data is provided in this article

**Table:** 

**Value of the Data** • These data on the effect of oral DBP exposure on uterine DNA methyltransferase activity provide insight into the mechanisms of action of this endocrine-disrupting chemical. • These data will benefit the fields of reproductive biology and toxicology by enhancing understanding of how environmentally-induced epigenetic alterations can affect normal uterine function, reproduction, and the development of several complex diseases that compromise women's health. • These data highlight the potential negative effects of phthalates on epigenetic regulation of uterine function; thus, they will stimulate the formulation of novel hypotheses and experiments aimed at elucidating the role of phthalate exposures on the development of epigenetically-driven uterine diseases.

## 1 Data description

**Fig. 1:**
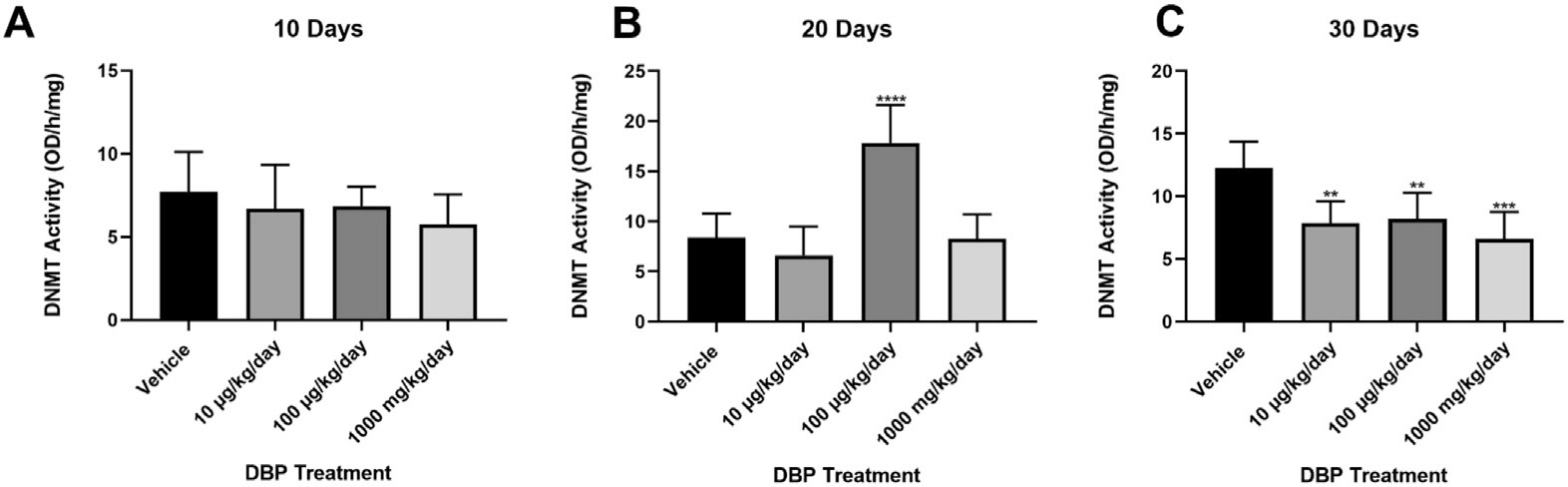
Effects of oral exposure to DBP on uterine DNA Methyltransferase (DNMT) activity (n = 8/treatment/timepoint; total n = 96). DNMT activity in uteri was expressed as the mean value ± standard error (SEM). CD-1 were dosed as described in Material and Methods for 10 days (**A**), 20 days (**B**), and 30 days (**C**) with DBP. Asterisks (*) indicate statistical differences versus vehicle (**p < 0.05), (***p < 0.001) and (****p < 0.0001).

In this report, we present data on the activity of uterine DNA methyltransferase in CD-1 mice treated with tocopherol-stripped corn oil (vehicle) or DBP dissolved in oil at 10 μg/kg/day, 100 μg/kg/day and 1000 mg/kg/day for 10, 20, and 30 days [[1], [2]]. DBP significantly disrupted the activity of DNMT at 20 and 30 days as compared with vehicle. These effects were not seen at 10 days. At 20 days of DBP exposure, we observed an increase on DNMT activity in the uteri of mice treated with DBP at 100 μg/kg/day. This effect of DBP exposure at 100 μg/kg/day was dose specific as it disappeared with increasing dose. Interestingly, at 30 days a reduction in DNMT activity was observed in all doses as compared with vehicle (Fig. 1). The raw data, terminal estrous cycle stage, and normalized values (OD and blank averages) of this study separated by time points are shown in Table 1, Table 2, Table 3.

**Table 1:** DNMT activity raw data: Treatment for 10 days with DBP.

Cycle	Treatment	Average OD 450	OD-Blank	DNMT Activity
M	vehicle	0.13695	0.10285	5.143
D	vehicle	0.14	0.1059	5.295
E	vehicle	0.25205	0.21795	10.898
D	vehicle	0.1472	0.1131	5.655
D	vehicle	0.24225	0.20815	10.408
D	vehicle	0.16095	0.12685	6.343
D	vehicle	0.19715	0.16305	8.153
D	vehicle	0.2306	0.1965	9.825
P	10 μg/kg/day	0.1844	0.1503	7.515
D	10 μg/kg/day	0.25385	0.21975	10.988
D	10 μg/kg/day	0.1917	0.1576	7.880
P	10 μg/kg/day	0.1152	0.0811	4.055
M	10 μg/kg/day	0.08655	0.05245	2.623
M	10 μg/kg/day	0.12895	0.09485	4.743
D	10 μg/kg/day	0.19465	0.16055	8.028
P	10 μg/kg/day	0.10865	0.07455	3.728
M	100 μg/kg/day	0.15415	0.12005	6.003
D	100 μg/kg/day	0.17895	0.14485	7.243
D	100 μg/kg/day	0.13555	0.10145	5.073
D	100 μg/kg/day	0.2035	0.1694	8.470
D	100 μg/kg/day	0.27495	0.24085	12.043
M	100 μg/kg/day	**	**	**
D	100 μg/kg/day	0.1747	0.1406	7.030
E	100 μg/kg/day	0.18055	0.14645	7.323
D	1000 mg/kg/day	0.27615	0.24205	12.103
D	1000 mg/kg/day	0.1894	0.1553	7.765
D	1000 mg/kg/day	0.15095	0.11685	5.843
D	1000 mg/kg/day	0.1087	0.0746	3.730
D	1000 mg/kg/day	0.1518	0.1177	5.885
P	1000 mg/kg/day	0.119	0.0849	4.245
D	1000 mg/kg/day	0.2034	0.1693	8.465
D	1000 mg/kg/day	0.12165	0.17045	8.523

** Tissue not available for the experiment.

**M**: Metestrus.

**D**: Diestrus.

**P**: Proestrus.

**E**: Estrus.

**Table 2:** DNMT activity raw data: Treatment for 20 days with DBP.

Cycle	Treatment	Average OD 450	OD-Blank	DNMT Activity
P	vehicle	0.20455	0.17045	8.523
M	vehicle	0.2678	0.2337	11.685
M	vehicle	0.2155	0.1814	9.070
D	vehicle	0.19685	0.16275	8.138
P	vehicle	0.2102	0.1761	8.805
M	vehicle	0.2076	0.1735	8.675
P	vehicle	0.21565	0.18155	9.078
E	vehicle	0.0943	0.0602	3.010
P	10 μg/kg/day	0.35985	0.32575	16.288
P	10 μg/kg/day	0.1349	0.1008	5.040
P	10 μg/kg/day	0.11535	0.08125	4.063
E	10 μg/kg/day	**	**	**
P	10 μg/kg/day	0.12395	0.08985	4.493
P	10 μg/kg/day	0.1491	0.115	5.750
E	10 μg/kg/day	0.24625	0.21215	10.608
P/E	10 μg/kg/day	0.2311	0.197	9.850
P	100 μg/kg/day	0.48345	0.48345	24.1725
P	100 μg/kg/day	0.3331	0.299	14.950
E	100 μg/kg/day	0.48105	0.44695	22.348
P/E	100 μg/kg/day	0.4348	0.4007	20.035
P	100 μg/kg/day	0.3736	0.3395	16.975
P	100 μg/kg/day	0.3704	0.3363	16.815
P	100 μg/kg/day	0.25755	0.22345	11.173
D	100 μg/kg/day	0.391	0.3569	17.845
P	1000 mg/kg/day	0.39015	0.35605	17.803
P	1000 mg/kg/day	0.19435	0.16025	8.013
P	1000 mg/kg/day	0.19235	0.15825	7.913
P/E	1000 mg/kg/day	0.28365	0.24955	12.478
P	1000 mg/kg/day	0.1734	0.1393	6.965
P	1000 mg/kg/day	0.19935	0.16525	8.263
E	1000 mg/kg/day	0.2299	0.1958	9.790
D	1000 mg/kg/day	0.1245	0.0904	4.520

** Tissue not available for the experiment.

**M**: Metestrus.

**D**: Diestrus.

**P**: Proestrus.

**E**: Estrus.

**Table 3:** DNMT activity raw data: Treatment for 30 days with DBP.

Cycle	Treatment	Average OD 450	OD-Blank	DNMT Activity
P	vehicle	0.2986	0.2645	13.225
P	vehicle	0.2794	0.2453	12.265
D	vehicle	0.22225	0.18815	9.408
D	vehicle	0.35055	0.31645	15.823
P	vehicle	0.28125	0.24715	12.358
P	vehicle	0.2869	0.2528	12.640
D	vehicle	0.2344	0.2003	10.015
D	vehicle	0.16235	0.12825	6.413
P	10 μg/kg/day	0.12785	0.09375	4.688
P	10 μg/kg/day	0.21725	0.18315	9.158
P	10 μg/kg/day	0.2204	0.1863	9.315
E	10 μg/kg/day	0.20195	0.16785	8.393
D	10 μg/kg/day	0.20645	0.17235	8.618
P/E	10 μg/kg/day	**	**	**
P	10 μg/kg/day	0.1763	0.1422	7.110
P	10 μg/kg/day	0.337	0.3029	15.145
E	100 μg/kg/day	0.2255	0.1914	9.570
P	100 μg/kg/day	0.2423	0.2082	10.410
P	100 μg/kg/day	0.232	0.1979	9.895
P	100 μg/kg/day	0.135	0.1009	5.045
P	100 μg/kg/day	0.35705	0.32295	16.148
E	100 μg/kg/day	0.35845	0.32435	16.218
P	100 μg/kg/day	0.18175	0.14765	7.383
E	100 μg/kg/day	0.17215	0.13805	6.903
P	1000 mg/kg/day	0.188	0.1539	7.695
E	1000 mg/kg/day	0.1359	0.1018	5.090
P	1000 mg/kg/day	0.1606	0.1265	6.325
P	1000 mg/kg/day	0.15935	0.12525	6.263
D	1000 mg/kg/day	0.1267	0.0926	4.630
E	1000 mg/kg/day	0.253	0.2189	10.945
P	1000 mg/kg/day	0.5554	0.5213	26.065
D	1000 mg/kg/day	0.1393	0.1052	5.260

** Tissue not available for the experiment.

**M**: Metestrus.

**D**: Diestrus.

**P**: Proestrus.

**E**: Estrus.

## 2 Experimental design, materials, and methods

### 2.1 Animal model

Female CD-1 mice (28 days old) were purchased from Charles River Laboratories (Charles River, CA). Animals were housed at the University of Illinois College of Veterinary Medicine Central Animal Facility with food and water provided at libitum, temperature set at 22 ± 1 °C, and 12L:12D cycles. Prior to entering the study, animals were allowed to acclimate to the animal facilities for at least 48 h. Animals were dosed as described below and euthanized by CO_2_ inhalation followed by cervical dislocation. The use of animals in these studies was approved by the University of Illinois Institutional Animal Care and Use Committee and conformed to the Guide for the Care and Use of Experimental Animals [3].

### 2.2 In vivo exposure to Dibutyl Phthalate (DBP) and tissue collection

On postnatal day 35, mice (n = 8/treatment; total = 32 mice per time point) were pipet-fed tocopherol-stripped corn oil (vehicle control; MP Biomedicals, Solon, OH) alone or dibutyl phthalate (99.6% purity, Sigma-Aldrich, St. Louis, MO) dissolved in oil at 10 μg/kg/day, 100 μg/kg/day, and 1000 mg/kg/day as previously described [4]. These doses were selected based on reported intake estimates in the general population (7–10 μg/kg/day) [5] and in medically and occupationally exposed subjects (up to 233 μg/kg/day) [2,6]. The highest dose of 1000 mg/kg/day was included to compare to exposure levels typically used in classical toxicity testing. Weights, estrous cyclicity, and overall health were recorded daily throughout the dosing period. At the end of 10, 20, or 30 days of daily oral dosing, animals were sacrificed within 1–2 hours after the final dose. The stage of the estrous cycle was recorded, and the uteri dissected, weighed and frozen.

### 2.3 Estrous cyclicity

Terminal estrous cycle stage was determined by vaginal smearing prior to euthanasia. Briefly, mice were restrained gently and 20 μL of sterile-filtered PBS was used to perform a vaginal washing. Vaginal washings were visualized unstained under an inverted microscope without knowledge of treatment [7].

### 2.4 Nuclear extractions

Frozen uteri were weighed and cut into small pieces (1–2 mm^3^) with a scalpel before homogenization with a Bullet Blender Storm 24 (Next Advance, Averill Park, NY). The homogenate was resuspended in lysis buffer containing 2% SDS in PBS and supplemented with proteinase and phosphatase inhibitors. Nuclear extracts were isolated using EpiQuik™ Nuclear Extraction Kit (Epigentek, Brooklyn, NY). Total protein concentration was quantified using Bio-Rad protein assay (Valencia, CA). BSA was used to generate a standard curve.

### 2.5 DNMT activity

EpiQuik DNMT Activity/Inhibition ELISA Easy Kit™ immunoassay (Epigentek, Brooklyn, NY) was used to measure total DNMT activity using nuclear extracts from frozen uterine tissues. This assay provides information on global DNMT activity including DNMTs 1, 3A, 3B, 2 and 3L without distinction. In brief, nuclear extracts (20 μg) were added to the wells on the EpiQuik DNMT Activity/Inhibition ELISA Easy Kit™ immunoassay 96-well plate and incubated for 1 hour according to the manufacturer's instructions. Colorimetric analysis was conducted using a microplate reader. The ratio of methylated DNA, which is proportional to enzyme activity, was measured at a wavelength of 450 nm. The activity of DNMT enzymes is proportional to the optical density intensity measured. DNMT activity was calculated using the following formula:

$$DNMTActivity\;\langle OD\text{|}h\text{|}mg\rangle =\frac{Sample\;OD-Blank\;OD}{Protein\;amount\;ug\times hour\;}\times 1000$$

Two technical replicates were conducted for validation purposes.

### 2.6 Statistical analyses

Parametric analysis of variance (ANOVA) with Dunnett's post-test were conducted to determine statistical significance of differences among study groups using GraphPad Prism 8 (GraphPad Software, Inc., La Jolla, CA). Statistical significance was set at p < 0.05.
